# Longitudinal relationships between pet ownership and cognitive functioning in later adulthood across pet types and individuals’ ages

**DOI:** 10.1038/s41598-025-03727-9

**Published:** 2025-05-30

**Authors:** Adriana Rostekova, Charikleia Lampraki, Jürgen Maurer, Clément Meier, Maud Wieczorek, Andreas Ihle

**Affiliations:** 1 Department of Psychology, University of Geneva, Chemin de Pinchat 22, Carouge, Geneva, 1227 Switzerland; 2 Center for the Interdisciplinary Study of Gerontology and Vulnerability, University of Geneva, Geneva, Switzerland; 3 Swiss Centre of Excellence in Life Course Research LIVES, Lausanne and Geneva, Switzerland; 4 Faculty of Business and Economics, University of Lausanne, Lausanne, Switzerland; 5 Swiss Centre of Expertise in the Social Sciences (FORS), Lausanne, Switzerland; 6 Centre on Aging and Mobility, University of Zurich, Zurich, Switzerland

**Keywords:** Cognitive ageing, Executive functioning, Episodic memory, Pet ownership, Human-animal interaction, Old age, Psychology, Human behaviour

## Abstract

Pet ownership has been shown to have a positive influence on cognitive functioning and cognitive decline in late adulthood. However, there is limited understanding of how different species of pets are associated with these outcomes in different age groups. This study employed a multilevel modelling approach using data from eight waves of the Survey of Health and Retirement in Europe (SHARE) to longitudinally examine: (1) the relationship between pet ownership and cognitive decline over 18 years among adults aged 50 and older, (2) The moderating role of younger vs. older age subgroups on this relationship, and (3) the distinct role of owning dogs, cats, birds, and fish. The targeted cognitive domains were executive functioning and episodic memory. Results confirmed that pet ownership is associated with slower decline in both cognitive domains. While age group did not moderate this relationship, differences emerged in the relationship between cognitive decline and individual pet species. Dog and cat ownership was associated with slower cognitive decline compared to no pet ownership; bird and fish ownership showed no such association. These findings suggest that dog and cat ownership might act as a protective factor aiding to slow down cognitive decline, thus contributing to healthy cognitive ageing.

**Supplementary Information:**

The online version contains supplementary material available at 10.1038/s41598-025-03727-9.

## Introduction

Age-related cognitive decline is an increasingly pressing concern in public health, which may begin in early adulthood and accelerate with increasing age^1,2^. While much research is still investigating the precise mechanisms of cognitive ageing, previous studies have identified several contributing factors, including for example genetics, general health and lifestyle choices^3–7^. Cognitive decline is a major public health concern on both individual and societal levels due to its association with diminished well-being and health-related quality of life^8,9^ as well as increased caregiving burden^10^ and health- and long-term care costs^11^. Therefore, it is important to investigate potential avenues to support cognitive health across the lifespan.

With regards to individual lifestyle factors, previous research has shown that, for example, an adherence to a Mediterranean- or DASH-style diet rich in vegetables, fruits, whole grains and unsaturated fats; sleeping 7–8 h per night with limited night-time fragmentation; regular participation in social clubs or volunteering; enrolment in adult-education courses; or employment in cognitively demanding occupations, are related to reduced or delayed deterioration of cognitive functioning^12–14^. Another potentially protective factor of cognitive health, which has received so far relatively little attention, is pet ownership. Approximately 38% of Europeans own pets, with similar rates of pet ownership estimated in older adults aged 50 and above^14–17^.

Numerous studies have linked pet ownership to a range of physical and mental health benefits^18–20^. When it comes to pet ownership in later age specifically, it has been shown to have a positive effect on older adults’ well-being and feelings of companionship^19^ as well as on their physical health. A systematic review by Gee and Mueller^20^ found robust evidence linking pet ownership to better physical health, in particular, cardiac health and physiological responses to stress, such as lower blood pressure or lower risk of fatal cardiac events in patients with hypertension. Furthermore, the review revealed that pet ownership had important psychosocial benefits. Specifically, older pet owners perceived lower rates of loneliness, and dog ownership in particular was linked to increased physical activity, which in turn related to positive social outcomes.

A further systematic review by Hughes and colleagues^21^ similarly concluded that human-animal interactions can benefit older adults’ physical but also mental health, including improvements in their quality of life and levels of depression and anxiety. Additionally, they identified five studies suggesting that the human-animal interaction could improve cognitive health and eight studies which found no effect on cognitive functioning. Most of the included studies focused on animal-assisted interventions and the one identified study which focused on pet ownership specifically, albeit cross-sectional, found significantly better executive functioning in pet owners^22^, suggesting that long-term pet ownership might be associated with better cognitive health. The empirical evidence in this newly emerging field of research is still limited, often mixed, and primarily derived from studies involving animal-assisted interventions. However, as outlined in the following, there are several reasons to hypothesize that pet ownership may be associated with slower cognitive decline.

Firstly, an increase in physical activity and a decrease in loneliness are both factors known to be related to reduced risk and a slower rate of cognitive decline^23,24^ Secondly, pet ownership has also been associated with increased social interaction^25^ and reduced anxiety symptoms^26^. These factors, as highlighted in meta-analyses^27,28^ are linked to a lower risk of cognitive impairment in older adults. Thirdly, the presence of a companion animal has further been associated with reductions in stress levels through the decrease in cortisol levels and heart rate during stressful situations^29^. Finally, pet ownership appears to not only lower the blood pressure response to mental stress^30^ but has also been associated with lower systemic blood pressure^31^. In sum, these findings highlight the importance of maintaining low stress levels and weak physiological responses to stress (through the excessive cortisol production and the associated hippocampal damage), as they have been linked to reduced risk of cognitive impairment^32–35^ and to less steep cognitive decline^35–37^. Although pet ownership is linked to many factors that have been also related to reduced cognitive decline, there is still only limited longitudinal and population-level research regarding the direct link between pet ownership and cognitive outcomes.

There is cross-sectional evidence pointing towards pet ownership being associated with higher levels of cognitive performance in several areas including processing speed, attentional control or episodic memory^38,39^. In terms of longitudinal studies, the empirical evidence appears to be inconsistent and dependent on various factors. For example, Branson and Cron^40^ found no relationship between pet caretaking and the risk of developing a mild cognitive impairment over a 12-year timespan after adjusting for potential confounding factors in their study of 2251 older adults aged 50 and above. This could be due to the fact pet caretaking might entail limited exposure to pets compared to their ownership, and hence some of the pet ownerships’ benefits associated with effects on cognitive decline were not experienced by all participants.

Applebaum and colleagues^41^, on the other hand, found that participants aged 65 and older who owned pets for more than 5 years had higher mean composite cognitive scores compared to those who owned pets for a shorter period of time or not at all. Yet, they found no association between cognitive decline and pet ownership in adults aged between 50 and 65, suggesting that there might be age group differences in the association of pet ownership with cognitive functioning. Therefore, further and fine-grained investigations are necessary to further elucidate the role of age. Lastly, Li and colleagues^42^ found slower cognitive decline over 8 years in pet owners compared to non-owners in measures of verbal fluency and verbal memory. Their additional analysis showed that pet ownership was associated with a slower decline in these domains only in individuals living alone compared to those living with others.

To better understand the pattern of associations between pet ownership and cognitive decline, it would also be of interest to separately examine the contributions of owning different species of pets. For example, Friedmann and colleagues^43^ found, with modest to moderate effect sizes, that pet owners, and especially dog owners, experienced slower cognitive decline over ten years across various facets of cognitive functioning, such as memory, executive functioning, language, psychomotor speed, and processing speed, compared to non-owners. Cat owners showed less decline in memory and language functioning. Additionally, dog owners who regularly walked their dogs had slower cognitive decline than those who did not, suggesting the important role of physical activity through pet ownership for cognitive health. However, the fact that several domains of cognitive functioning showed slower deterioration also in cat owners and dog owners who did not walk their dogs, implies that other factors besides physical activity per se may be at play. Despite the potential recall bias, as the participants had to recall having a pet in the past, these findings highlight the need to further scrutinise in detail the associations of different pet species with cognitive decline.

To build on these previous findings and to overcome some of the remaining research gaps outlined above, the present study will first aim to investigate the association of overall pet ownership with cognitive decline over a period of 18 years in a large European population-based sample in order to achieve greater statistical power, higher precision, and greater generalisability, compared to previous studies. Additionally, we will also examine the potential moderating role of younger and older age groups as well as the specific associations with different pet types. Based on the empirical literature outlined above, we hypothesize that: (1) Pet ownership will be associated with slower cognitive decline, (2) Older age groups will have a stronger association between pet ownership and reduced cognitive decline than younger ones, and (3) There will be species-specific differences in the strength of associations between the pet ownership and cognitive decline with larger associations for dog and cat owners compared to owners of pet resulting in less deep human-animal interactions.

## Results

The participants were aged between 50 and 99 with a mean (SD) age of 63.4 (9.5). 53.5% of the participants were women. Of the final sample, 39.4% of participants were pet owners. Further details on the characteristics of the sample are presented in Supplementary Table S1.

### Cognitive decline as a function of pet ownership

At baseline, pet owners in our sample demonstrated higher scores of verbal fluency but lower scores of immediate and delayed recall at the baseline in comparison to non-owners (see Table 1 for full results). Time was significantly associated with a decline in all three cognitive scores. With a one-unit increase in time, which indicates a two-year difference in our study, verbal fluency scores decreased by β = -0.34 (95%CI [-0.37, -0.31]). A similar decrease was found in immediate and delayed recall scores, signifying a general decline in cognitive performance over time in the sample, independent of pet ownership status and other covariates.

**Table 1:** The relationship between pet ownership, time and cognitive functioning.

Predictors	Verbal fluency score		Immediate recall score		Delayed recall score	
	Estimates [CI]	p-value	Estimates [CI]	p-value	Estimates [CI]	p-value
(Intercept)	20.80 [20.50, 21.09]	< 0.001	4.99 [4.92, 5.05]	< 0.001	3.44 [3.37, 3.52]	< 0.001
Time	– 0.34 [– 0.37, – 0.31]	< 0.001	– 0.08 [– 0.09, – 0.08]	< 0.001	– 0.08 [– 0.09, – 0.07]	< 0.001
Pet ownership	0.33 [0.15, 0.51]	< 0.001	– 0.04 [– 0.08, – 0.00]	0.035	– 0.08 [– 0.13, – 0.04]	< 0.001
Pet ownership x time	0.11 [0.07, 0.16]	< 0.001	0.03 [0.02, 0.04]	< 0.001	0.03 [0.02, 0.05]	< 0.001
Age	– 0.15 [– 0.16, – 0.14]	< 0.001	– 0.05 [– 0.05, – 0.05]	< 0.001	– 0.06 [– 0.07, – 0.06]	< 0.001
Sex	0.11 [– 0.07, 0.29]	0.217	0.35 [0.31, 0.38]	< 0.001	0.45 [0.41, 0.50]	< 0.001
Education level	– 3.69 [– 3.87, – 3.51]	< 0.001	– 0.79 [– 0.83, – 0.76]	< 0.001	– 0.80 [– 0.84, – 0.75]	< 0.001
Moderate physical activity	– 0.88 [– 0.97, – 0.78]	< 0.001	– 0.16 [– 0.18, – 0.14]	< 0.001	– 0.16 [– 0.18, – 0.13]	< 0.001
Chronic comorbidities	– 0.19 [– 0.26, – 0.13]	< 0.001	– 0.05 [– 0.07, – 0.04]	< 0.001	– 0.06 [– 0.08, – 0.05]	< 0.001
Living alone	0.03 [– 0.20, 0.26]	0.809	– 0.09 [– 0.13, – 0.04]	< 0.001	– 0.06 [– 0.12, 0.00]	0.057
Marginal R^2^	0.147		0.187		0.165	
Conditional R^2^	0.622		0.498		0.533	

**Fig. 1:**
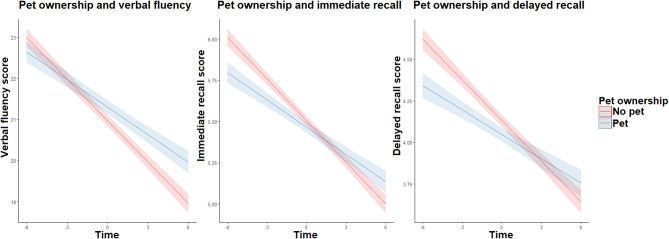
The relationship between pet ownership and cognitive decline.

However, upon examining the interaction between pet ownership and time, the association between time and verbal fluency score became positive for pet owners, β = 0.11 (95%CI [0.07, 0.16]), with a similar shift found in immediate and delayed recall scores (see Fig. 1). This suggests that while cognitive performance worsened for all participants over time, the decline was less pronounced in pet owners. Therefore, whilst pet owners performed better on verbal fluency but worse on recall tasks at baseline compared to non-owners, over time, their cognitive decline was slower (see Table 1 for details).

### Cognitive decline as a function of age group and pet ownership

Overall, age group did not moderate the relationship between pet ownership and cognitive decline in any of the three measured cognitive outcomes. An inclusion of age group in the verbal fluency model significantly improved the model fit (see Supplementary Table S2). The main effect of the age group was statistically significant, with participants in the younger age group scoring higher in verbal fluency than the participants in the older age group at the baseline. However, both two-way and three-way interactions with pet ownership and time yielded insignificant results, suggesting that the relationship between pet ownership and cognitive decline did not differ by age group (see Table 2).

**Table 2:** The relationship between pet ownership, age category, time and cognitive functioning.

Predictors	Verbal fluency score	
	Estimates [CI]	p-value
(Intercept)	19.37 [19.06, 19.69]	< 0.001
Time	– 0.60 [– 0.65, – 0.56]	< 0.001
Pet ownership	0.29 [0.02, 0.57]	0.036
Age group	2.27 [2.04, 2.50]	< 0.001
Pet ownership x time	0.10 [0.02, 0.18]	0.012
Pet ownership x age group	0.31 [– 0.05, 0.68]	0.091
Pet ownership x age group x time	– 0.07 [– 0.16, 0.03]	0.170
Sex	0.18 [– 0.00, 0.35]	0.052
Education level	– 3.82 [– 4.00, – 3.64]	< 0.001
Moderate physical activity	– 0.95 [– 1.04, – 0.86]	< 0.001
Chronic comorbidities	– 0.27 [– 0.33, – 0.20]	< 0.001
Living alone	– 0.29 [– 0.52, – 0.06]	0.013
Marginal R^2^	0.149	
Conditional R^2^	0.626	

Furthermore, adding the age group to the immediate and delayed recall models did not improve the model fit; we therefore excluded these models from further investigation of age group effects (see Supplementary Table S2).

### Cognitive functioning and decline as a function of specific pet species ownership

We further examined the role of specific pet species ownership on cognitive performance and decline.

**Fig. 2:**
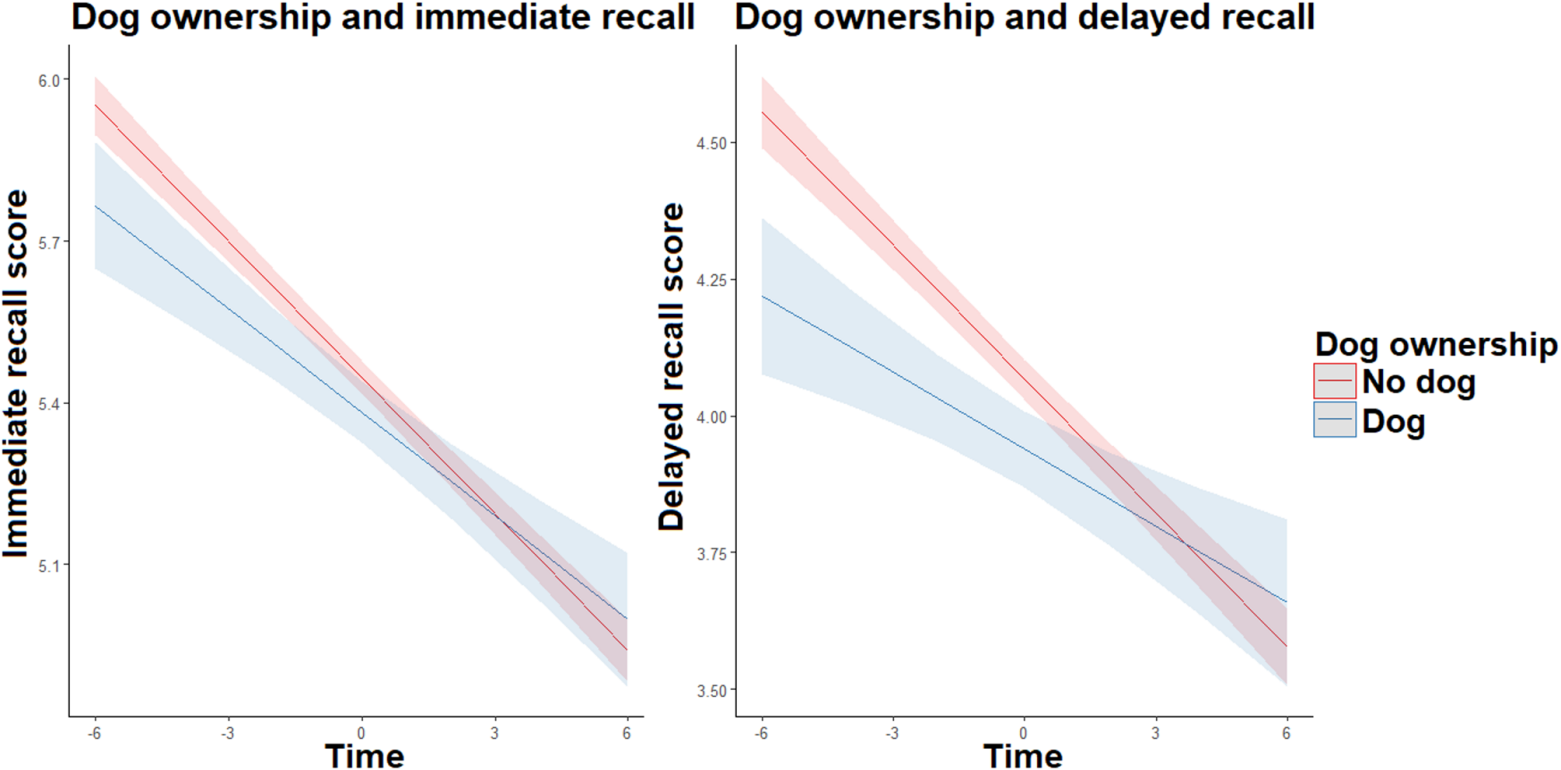
The relationship between dog ownership and cognitive decline.

At baseline, dog owners scored lower than people without pets in both immediate and delayed recall. Despite the negative effect of time on these scores, the interaction between dog ownership and time showed a positive relationship with these two scores, thus suggesting a slower decline in memory over time in dog owners in comparison with non-owners (see Fig. 2). However, including dog ownership in the verbal fluency model did not improve the model fit, indicating that dog ownership did not meaningfully contribute to explaining the variability in the decline in verbal fluency over time (see Table 3 for full results).

**Table 3:** The relationship between dog ownership, time and cognitive functioning.

Predictors	Immediate recall score		Delayed recall score	
	Estimates [CI]	p-value	Estimates [CI]	p-value
(Intercept)	4.95 [4.88, 5.02]	< 0.001	3.40 [3.31, 3.49]	< 0.001
Time	– 0.08 [– 0.09, – 0.08]	< 0.001	– 0.08 [– 0.09, – 0.07]	< 0.001
Dog ownership	– 0.06 [– 0.12, – 0.01]	0.030	– 0.13 [– 0.20, – 0.06]	< 0.001
Dog ownership x time	0.02 [0.00, 0.04]	0.040	0.03 [0.01, 0.06]	0.004
Age	– 0.05 [– 0.05, – 0.05]	< 0.001	– 0.06 [– 0.07, – 0.06]	< 0.001
Sex	0.33 [0.29, 0.37]	< 0.001	0.44 [0.38, 0.49]	< 0.001
Education level	– 0.77 [– 0.81, – 0.73]	< 0.001	– 0.78 [– 0.83, – 0.72]	< 0.001
Moderate physical activity	– 0.15 [– 0.18, – 0.13]	< 0.001	– 0.17 [– 0.19, – 0.14]	< 0.001
Chronic comorbidities	– 0.05 [– 0.06, – 0.03]	< 0.001	– 0.06 [– 0.08, – 0.04]	< 0.001
Living alone	– 0.08 [– 0.13, – 0.03]	0.003	– 0.05 [– 0.12, 0.01]	0.119
Marginal R^2^	0.183		0.165	
Conditional R^2^	0.500		0.535	

**Fig. 3:**
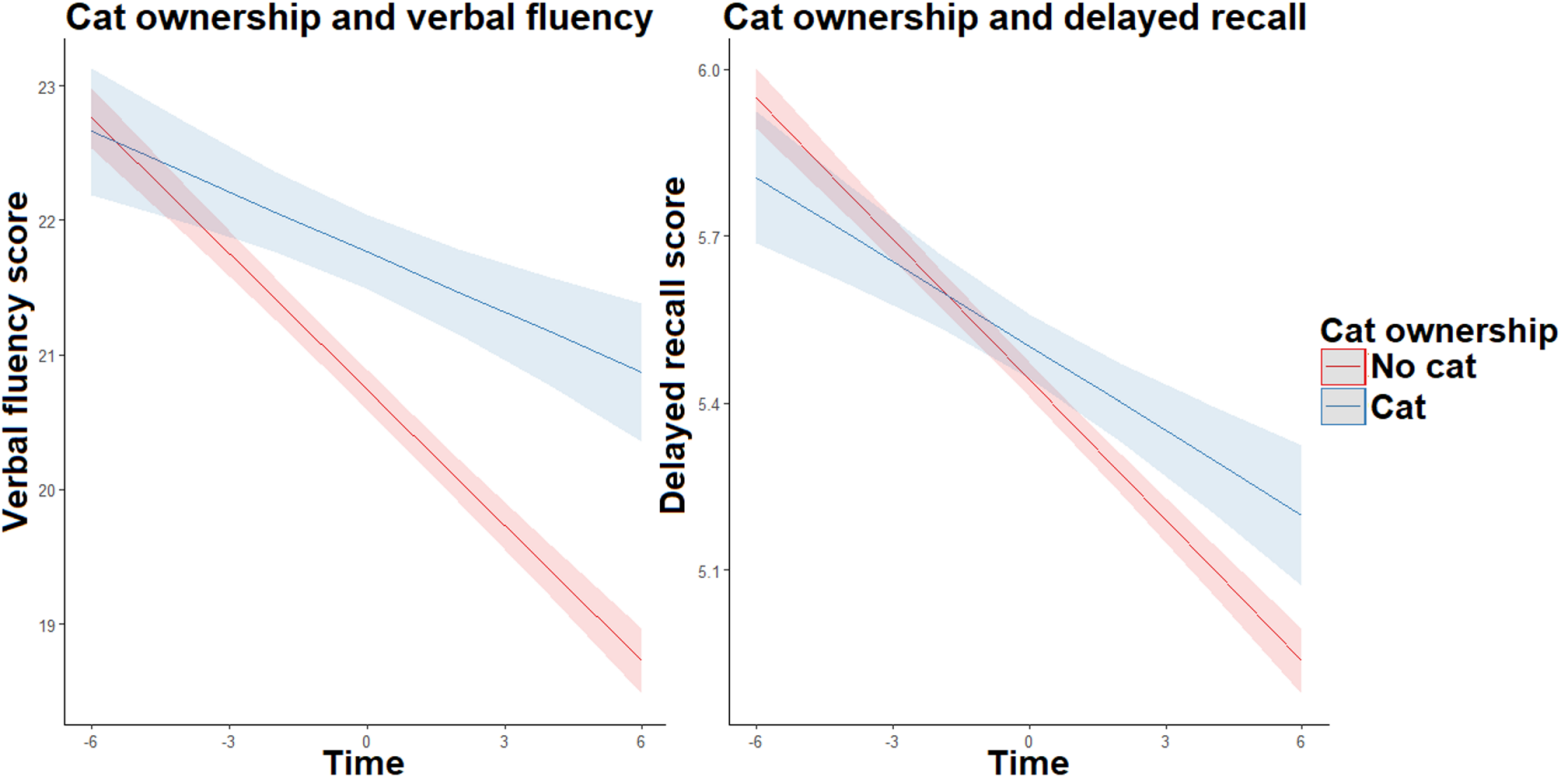
The relationship between cat ownership and cognitive decline.

Cat owners scored higher than non-owners at baseline in measures of verbal fluency and delayed recall. An inclusion of cat ownership in the interaction term with time had also a significantly positive relationship with these scores, indicating that cat owners experienced a slower decline in these cognitive domains compared to non-owners (see Fig. 3). However, including cat ownership in the immediate recall model did not improve the model fit, implying that cat ownership did not meaningfully aid in explaining the variability in the decline of immediate recall over time (see Table 4 for full results).

**Table 4:** The relationship between cat ownership, time and cognitive functioning.

Predictors	Verbal fluency score		Delayed recall score	
	Estimates [CI]	p-value	Estimates [CI]	p-value
(Intercept)	20.57 [20.22, 20.91]	< 0.001	3.35 [3.27, 3.44]	< 0.001
Time	– 0.34 [– 0.37, – 0.31]	< 0.001	– 0.08 [– 0.09, – 0.07]	< 0.001
Cat ownership	1.02 [0.74, 1.31]	< 0.001	0.09 [0.01, 0.16]	0.019
Cat ownership x time	0.19 [0.11, 0.26]	< 0.001	0.05 [0.03, 0.07]	< 0.001
Age	– 0.15 [– 0.16, – 0.14]	< 0.001	– 0.06 [– 0.07, – 0.06]	< 0.001
Sex	0.11 [– 0.10, 0.32]	0.288	0.46 [0.41, 0.51]	< 0.001
Education level	– 3.53 [– 3.74, – 3.32]	< 0.001	– 0.76 [– 0.21, – 0.15]	< 0.001
Moderate physical activity	– 0.93 [– 1.04, – 0.82]	< 0.001	– 0.18 [– 0.21, – 0.15]	< 0.001
Chronic comorbidities	– 0.22 [– 0.30, – 0.14]	< 0.001	– 0.06 [– 0.08, – 0.04]	< 0.001
Living alone	0.00 [– 0.26, 0.26]	0.990	– 0.07 [– 0.14, – 0.01]	0.026
Marginal R^2^	0.147		0.168	
Conditional R^2^	0.612		0.537	

At baseline, bird owners scored lower than non-owners across all three measured cognitive domains. However, the interaction between bird ownership and time did not significantly improve the model fit in any of the three models, suggesting that bird ownership does not meaningfully explain the variability in cognitive decline (see Table 5 for full results).

**Table 5:** The relationship between bird ownership and cognitive functioning.

Predictors	Verbal fluency score		Immediate recall score		Delayed recall score	
	Estimates [CI]	p-value	Estimates [CI]	p-value	Estimates [CI]	p-value
(Intercept)	20.74 [20.38, 21.09]	< 0.001	4.92 [4.85, 5.00]	< 0.001	3.35 [3.26, 3.44]	< 0.001
Time	– 0.34 [– 0.37, – 0.31]	< 0.001	– 0.08 [– 0.09, – 0.08]	< 0.001	– 0.08 [– 0.09, – 0.07]	< 0.001
Bird ownership	– 1.35 [– 1.88, – 0.81]	< 0.001	– 0.32 [– 0.43, – 0.20]	< 0.001	– 0.40 [– 0.53, – 0.26]	< 0.001
Age	– 0.14 [– 0.15, – 0.13]	< 0.001	– 0.05 [– 0.05, – 0.05]	< 0.001	– 0.06 [– 0.07, – 0.06]	< 0.001
Sex	– 0.04 [– 0.26, 0.18]	0.723	0.33 [0.28, 0.37]	< 0.001	0.44 [0.38, 0.49]	< 0.001
Education level	– 3.56 [– 3.79, – 3.34]	< 0.001	– 0.77 [– 0.82, – 0.73]	< 0.001	– 0.75 [– 0.81, – 0.70]	< 0.001
Moderate physical activity	– 0.93 [– 1.04, – 0.81]	< 0.001	– 0.16 [– 0.18, – 0.13]	< 0.001	– 0.17 [– 0.20, – 0.14]	< 0.001
Chronic comorbidities	– 0.21 [– 0.29, – 0.12]	< 0.001	– 0.05 [– 0.07, – 0.03]	< 0.001	– 0.06 [– 0.08, – 0.04]	< 0.001
Living alone	0.01 [– 0.26, 0.28]	0.952	– 0.08 [– 0.14, – 0.02]	0.006	– 0.05 [– 0.12, 0.02]	0.130
Marginal R^2^	0.144		0.186		0.163	
Conditional R^2^	0.609		0.502		0.540	

Lastly, an inclusion of fish ownership in the models did not improve the model fit for any of the cognitive outcomes, suggesting that fish ownership did not meaningfully contribute to explaining the variability in baseline cognitive scores as well as in cognitive decline.

### Cognitive decline as a function of age group and specific pet species pet ownership

An addition of age group to the interaction term for any pet species models did not significantly improve the model fit, indicating that the difference between age groups did not meaningfully contribute to explaining the variability in the relationship between specific pet species ownership and cognitive performance and decline (see Supplementary Tables S3-S6).

## Discussion

This study set out to resolve the inconsistent findings on the association between pet ownership and cognitive functioning and decline by examining the moderating roles of age group and pet species. Our results revealed that compared to participants without pets, pet owners exhibited higher baseline levels of verbal fluency but lower baseline levels of immediate and delayed recall. Despite this initial disadvantage, pet owners in our study also experienced slower cognitive decline across all three of these measures, thus supporting our hypothesis. The findings align with previous research on the relationship between pet ownership and cognitive functioning and cognitive decline in older adults^38,42,43^. Our study then adds to the past literature by investigating pet ownership in a large population-based European sample with data spanning almost two decades long, in contrast to previous studies that used smaller sample sizes and for shorter periods of time.

With regards to another key novelty of the present study, that is, the investigation of the individual contribution of each pet species to the relationship between pet ownership and cognitive decline, we found notable differences between the species. Both cat and dog owners experienced slower decline in multiple cognitive domains - dog owners in immediate and delayed recall, cat owners in verbal fluency and delayed recall. This was evident even in dog owners, who showed worse baseline memory performance in both recall measures. On the other hand, fish and bird ownership had no significant association with cognitive decline. This suggests that the overall effect of pet ownership on cognitive decline may be driven primarily by cat and dog ownership, rather than pet ownership in general, and therefore the specific pet species an individual owns influences its possible relationship with changes in cognition.

Several explanations may help explain the absence of this association in fish and bird owners, despite the reports of their ownership’ positive influence on wellbeing through factors such as companionship and verbal interactions^44^, strong attachment to the pet^45^ and reduced loneliness^46^ linked to bird ownership or improvements in mood, pain, relaxation, nutritional intake and bodyweight^47^ as well as stress reduction^48,49^ linked to fish ownership, all factors associated with cognitive benefits through mechanisms such as reduction in anxiety levels^28^, stress levels^35^, pain levels^50^ or body weight^51^.

Firstly, the evidence was scarce and generally came from small-sample studies, with an additional lack of recent research. There are also other reasons why these pet species may not be associated with the expected cognitive benefits. For example, the limited level of emotional support and frequently having to deal with the pet’s death due to its short lifespan may potentially limit the level of emotional connection one is able to develop with the pet fish^49^. Bird ownership may negatively affect the owner’s sleep quality due to the increased noise levels^44^, which has been shown to be associated with cognitive decline as well^52–54^. Therefore, the owners of these pets may not be able to fully experience the cognitive benefits associated with other types of pets, such as dogs and cats.

It is further possible that interaction with dogs and cats provides unique cognitive stimulation which may be less pronounced in other, less demanding pets. While the research is so far relatively scarce, there has been evidence of an increase in prefrontal brain activation and thus stronger attentional processes and emotional arousal caused by interaction with a dog compared to a non-living stimulus^55^ or of increased activation of the prefrontal cortex and the inferior frontal gyrus when interacting with cats, speculated to be linked to the characteristic, hard-to-predict temperament of cats^56^.

Secondly, there is also a possibility of increased social stimulation facilitated by these two pet species which may be linked to the slower cognitive decline experienced by their owners. Firstly, pet ownership could facilitate the creation of social support networks with others^57^, for example due to an increased frequency of social interactions when accompanied by a dog^58^. Additionally, the pet itself could act as an extension of one’s social network. Stammbach and colleagues^59^, for example, found that in strongly attached cat owners, their pets acted as additional sources of emotional support or even a substitute for a social network. Pets can further act as sources of support especially for people with limited or difficult relationships with their social network^60^ and have been shown to act as such also during times when social contact between people was limited, such as during the COVID-19 pandemic^61^. As such, it may be speculated that pets could potentially act as a part of one’s relational reserve which might then interact with cognitive reserves and thus contribute to slowing down cognitive decline^62^.

Finally, the age group analysis revealed that in our sample, age did not moderate the relationship between pet ownership and cognitive functioning or decline in any domain of cognitive functioning, both in the models of general pet ownership as well as of ownership of specific pet species. These findings contrast with those of Applebaum and colleagues^41^ who reported an association between sustained pet ownership and the mean composite cognitive scores (calculated from immediate and delayed recall, serial 7’s task and backwards count scores) only in participants aged 65 and over. Conversely, participants in our sample seem to have experienced a similar association between pet ownership and cognitive decline regardless of age group.

Despite the study’s novel insights with regards to longitudinal associations between the ownership of individual pet species and cognitive functioning, the present findings remain correlational. Whilst we did control for certain characteristics which tend to differentiate pet owners from non-owners, such as physical health or their physical activity levels, it remains plausible that the observed associations could be attributed also to selection effects. Pet owners differ systematically from those without pets, including differences between owners of specific pet species, such as dogs and cats^63^. Many of these characteristics (e.g., a higher prevalence of depression or anxiety) are themselves associated with faster cognitive decline, irrespective of pet ownership. Such factors, such as the aforementioned lower mental wellbeing, may also explain the lower baseline memory levels in pet owners seen in our data, although despite our large sample and the fact that a similar baseline pattern has been observed previously in another longitudinal cohort^43^, it cannot be ruled out that this discrepancy was found in our data by chance.

There are also certain other limitations which could be addressed by future research. As pet ownership was only assessed in the first wave of SHARE, it would be beneficial for cohort studies to include this variable at every assessment to enable the testing of the association between sustained pet ownership and cognitive decline. Furthermore, the current study focused exclusively on European participants. Future studies might therefore be able to confirm whether these findings hold in a global context, or whether there are perhaps cultural factors influencing the relationship between pet ownership and cognitive development in older adults. Such investigations could also potentially uncover further insights into the underlying mechanisms driving this association.

Overall, the present results suggest that pet ownership is associated with slower cognitive decline in older adults, and while this association is not dependent on their age group, it does differ depending on the specific pet species owned, with effects present in dog and cat owners as opposed to those owning birds and fish. These findings may therefore stimulate future investigation into the potential mechanisms driving these effects, for example via an in-depth exploration of the cognitive and social stimulation associated with dog and cat ownership, in particular with regard to the close personal bond that owners seem to form with these pet types, and how they can help support cognitive resilience in ageing populations. Such findings could then potentially help inform healthy ageing policies by advocating for measures such as financial assistance for expenses like veterinary care or pet insurance in order to make pet ownership more accessible to older adults, or an advocacy for animal-friendly senior housing options, such as assisted living facilities or nursing homes.

## Methods

### Sample

The dataset used for this study comprises eight waves of the Survey of Health, Ageing and Retirement in Europe (SHARE), a multidisciplinary and international panel database of longitudinal data collected biennially from individuals aged 50 years and above as well as their partners^64,65^. The currently available survey waves span 2004 to 2022, providing 18 years of data. Data collection is conducted via face-to-face computer-assisted personal interviewing (CAPI), which is then complemented by paper-and-pencil drop-off questionnaires. In accordance with the Declaration of Helsinki for research involving humans, SHARE was reviewed and approved by the Ethics Committee of the University of Mannheim, the Ethics Council of the Max Planck Society and country-specific ethics committees or institutional review boards when required. All participants provided informed consent.

This study included all SHARE waves from wave one to wave nine with the exception of wave three, in which cognitive functioning was not assessed, and utilised data from eleven European countries (Austria, Belgium, Denmark, France, Germany, Greece, Italy, the Netherlands, Spain, Sweden, and Switzerland). Included are the participants who took part in the first wave of data collection in 2004 and responded to the drop-off questionnaire which included questions regarding pet ownership. Additionally, 1369 participants who reported having been diagnosed with Parkinson’s disease, Alzheimer’s disease or other forms of dementia, organic brain syndrome, senility, or any other serious memory impairment at any point during their participation in the survey were excluded from the present study, as these individuals are likely to follow a different trajectory of cognitive decline compared to those without such impairments. Furthermore, we excluded participants who claimed to have a pet but did not select any of the offered pet categories (i.e. dog, cat, bird, fish or other). Finally, only complete cases (i.e., observations without missing information in any variable used in the analysis) were retained. Based on these criteria, the main analysis was conducted with a sample of 16,582 individuals.

To investigate the relationship between the ownership of specific pet species and cognitive decline, species-specific subsets of analyses were conducted separately for each of the four specified pet species. Participants who owned more than one pet species were excluded to ensure species-specific comparisons. Similarly, participants who reported owning a pet categorized as ‘other’ (not among the four specified species) were excluded, as SHARE did not request the participants to characterise those species. This resulted in the following sample sizes for the sub-analyses: 1,886 dog owners compared to 10,049 participants without pets, 1,778 cat owners compared to 10,049 participants without pets, 447 bird owners to 10,049 participants without pets, and 266 fish owners compared to 10,049 participants without pets.

### Measures

#### Pet ownership

Pet ownership was assessed in wave 1 using a self-administered paper-and-pencil drop-off questionnaire. Participants were asked “Do you currently have one or more of the following pets in your household?” with the options: dog, cat, bird, fish, other pets, and no pets in the household. The precise number of pets was not assessed. A binary variable was created with 0 = non-pet-owner and 1 = pet-owner. In addition, we created four binary variables for each pet type with 0 = non-pet-owner and 1 = dog owner, cat owner, bird owner or fish owner, respectively.

#### Cognitive functioning

Cognitive functioning was measured using computer-assisted personal interviewing (CAPI). The two domains of cognitive functioning assessed were episodic memory and executive functioning.

Episodic memory was measured using immediate and delayed free-recall tasks requiring participants to recall a ten-word list immediately, and again approximately ten minutes after the first assessment^66^. Executive functioning was assessed using a verbal fluency task, in which participants had to list as many words as possible from a specific semantic category, i.e. animals, within one minute^67^. All these measures were consistently assessed across all included waves. Higher values indicated higher cognitive functioning in all cognitive domains.

#### Age group

For the age group moderation analysis, participants were divided into two age groups based on the median split - those aged 62 and below versus those aged 63 and above at the time of the first assessment during wave 1. A median split was used to ensure comparable group sizes and to facilitate a straightforward comparison between the age categories.

#### Control variables

Several control variables, assessed using CAPI, were included in the study: age, sex (1 = male, 2 = female), education level (dichotomized into lower ISCED levels 1–2 vs. higher ISCED levels 3–6), physical activity level (measured via self-reported frequency of physical activity requiring moderate level of energy), number of chronic comorbidities, and living situation (determined based on whether the reported number of household residents was one vs. more; 0 = living with others and 1 = living alone). The values for these control variables were taken from wave 1, aligning with when pet ownership was reported.

### Analytical approach

Given the hierarchical structure of the longitudinal data, individual observations were nested within participants. We, therefore, used a multilevel modelling approach. To enhance the interpretability of the results, we applied cluster-centering by centering the time variable within each individual. This ensured that the intercepts represented each participant’s baseline score, i.e., their score at the start of their observations. This approach prevents conflating changes over time within individuals with differences in participants’ starting points. We further grand-mean centred age, moderate activity level and a number of chronic diseases, thus enabling the intercept to represent the average outcome score for a participant with an average level in these variables; the coefficients for these variables now reflect the deviations from the overall sample means.

First, we built an empty model for each cognitive outcome to estimate the amount of variability between individual participants and calculated the intraclass correlation coefficient (ICC) and the design effect (DEFF). The ICC was 0.59, 0.46 and 0.49 for the empty models fitted for verbal fluency, immediate recall and delayed recall respectively, meaning that 59%, 46% and 49% of the variance in the scores were explained by the variability between the participants. The DEFFs were above 1.5 (2.48, 2.16 and 2.22 respectively), thus warranting the use of a multilevel modelling approach.

Next, we built several intermediate models. Firstly, we introduced the fixed effects for covariate predictors, followed by a time-adjusted model with time added as a fixed effect in order to evaluate whether the cognitive performance changes over time assuming a consistent effect of time across individuals. This was followed by fitting an augmented model into which a random slope for time was added, allowing for heterogeneity in the participants’ rate of change over time and hence enabling us to observe different trajectories of cognitive decline over time across the participants.

The following model included pet ownership as a predictor to test whether it is associated with cognitive outcomes. Lastly, the final models included an interaction term between time and the pet ownership variable, thus testing whether the rate of change in the cognitive outcomes differed depending on the pet ownership status, testing our hypothesis that pet ownership will be associated with a lower cognitive decline. To test for the possibly differential effects of age group, the very last step consisted of adding age group into the aforementioned interaction term (time*pet ownership*age group). All models were tested using the Restricted Maximum Likelihood estimation method. This procedure was repeated for each cognitive outcome (verbal fluency score, immediate recall score and delayed recall score) separately for each pet species (pet, dog, cat, bird and fish ownership).

With the addition of each parameter, we compared the new model to the previous one using the Akaike and the Bayesian Information Criterion (AIC and BIC) and the − 2 log likelihood (-2LL) fit indices in order to assess whether the improvement in the fit is statistically significant and hence whether the adjustments improve model’s explanatory power, aiming to select the most parsimonious model (see Tables S2-S6 for more details on the model comparisons). To facilitate model fit comparisons, all models were refitted using Maximum Likelihood (ML) methods.

The models presented in the Results section are then the ones selected based on statistically significant improvements in fit, ensuring a balance between explanatory power and parsimony.

All analyses were conducted using the ‘lme4’ package in R^68,69^.

## Electronic supplementary material

Below is the link to the electronic supplementary material.

Supplementary Material 1

## Data Availability

SHARE data is available free of charge for scientific purposes to anyone with scientific affiliation after an individual registration. Detailed information regarding the application process can be accessed on the website share-eric.eu.
