# Supplementary material for: Longitudinal relationships between pet ownership and cognitive functioning in later adulthood across pet types and individuals’ ages
Source: Sci Rep. 2025 May 30;15:19066. doi: 10.1038/s41598-025-03727-9 (PMC12125269; doi:10.1038/s41598-025-03727-9)
Supplement: Supplementary file 1 — Supplementary Material 1 [file 41598_2025_3727_MOESM1_ESM.pdf]

Supplementary Table S1

Characteristics of the participants.

Characteristics	Total (%)
Age at wave 1 (Mean, SD)	63.42 (9.5)
Pet ownership	
Yes	6533 (39.4)
No	10049 (60.6)
Species distribution among owners †	
Dog	1886 (43.1)
Cat	1778 (40.6)
Bird	447 (10.2)
Fish	266 (6.1)
Age group	
Younger (≤62 at wave 1)	8722 (52.6)
Older (63≥ at wave 1)	7860 (47.4)
Sex	
Male	7705 (46.5)
Female	8877 (53.5)
Education level	
Lower (ISCED 1-2)	7799 (47.0)
Higher (ISCED 3-6)	8783 (53.0)
Moderate activity level	
More than once a week	11973 (72.2)
Once a week	2184 (13.2)
One to three times a month	793 (4.8)
Hardly ever, or never	1632 (9.8)
Number of chronic comorbidities	
0	4478 (27.0)
1	5407 (32.6)
2	3453 (20.8)
3	1835 (11.1)
4	840 (5.1)
5 and more	569 (3.5)
Living alone	
Yes	3177 (19.2)
No	13405 (80.8)

† Percentages are based on the number of pet owners included in the species-specific analysis (*n* = 4, 377), thus excluding participants who owned multiple pet species or pets classified as “other.”

Supplementary Table S2

Comparison of the pet ownership models.

	k	AIC	BIC	LL	Deviance	χ^2	*df*	p-value
**Verbal fluency models**								
Empty model	3	395355	395382	-197674	395349			
+ Covariates	9	391476	391558	-195729	391458	3890.42	6	<.001
+ Time effects	10	390739	390830	195360	390719	738.81	1	<.001
+ Random slope for time	12	390178	390287	-195077	390154	564.95	2	<.001
+ Pet ownership as a predictor	13	390170	390287	-195072	390144	10.62	1	.001
+ Interaction with time	14	390149	390275	-195060	390121	23.28	1	<.001
+ Interaction with age category	17	390093	390246	-195029	390059	62.00	3	<.001
**Immediate recall models**								
Empty model	3	225531	225558	-112763	225525			
+ Covariates	9	219591	219673	-109787	219573	5951.70	6	<.001
+ Time effects	10	218882	218972	-109431	218862	711.60	1	<.001
+ Random slope for time	12	218448	218557	-109212	218424	437.36	2	<.001
+ Pet ownership as a predictor	13	218445	218562	-109209	218419	5.67	1	.017
+ Interaction with time	14	218425	218551	-109198	218397	22.07	1	<.001
+ Interaction with age category	17	219003	219156	-109484	218969	0.00	3	1
**Delayed recall models**								
Empty model	3	244982	245009	-122488	244976			
+ Covariates	9	239837	239918	-119909	239819	5156.77	6	<.001
+ Time effects	10	239394	239485	-119687	239374	444.56	1	<.001
+ Random slope for time	12	238500	238609	-119238	238476	897.94	2	<.001
+ Pet ownership as a predictor	13	238486	238604	-119230	238460	16.13	1	<.001
+ Interaction with time	14	238470	238596	-119221	238442	18.61	1	<.001
+ Interaction with age category	17	390093	390246	-195029	390059	0.00	3	1

Supplementary Table S3
Comparison of the dog ownership models.

	k	AIC	BIC	LL	Deviance	χ^2	*df*	p-value
**Verbal fluency models**								
Empty model	3	278404	278430	-139199	278398			
+ Covariates	9	275735	275813	-137859	275717	2681.24	6	<.001
+ Time effects	10	275101	275188	-137541	275081	636.17	1	<.001
+ Random slope for time	12	274695	274799	-137335	274671	410.22	2	<.001
+ Dog ownership as a predictor	13	274697	274810	-137335	274671	0.00	1	.965
**Immediate recall models**								
Empty model	3	158768	158794	-79381	158762			
+ Covariates	9	154653	154731	-77317	154635	4127.26	6	<.001
+ Time effects	10	154031	154118	-77006	154011	623.40	1	<.001
+ Random slope for time	12	153706	153810	-76841	153682	329.37	2	<.001
+ Dog ownership as a predictor	13	153703	153815	-76838	153677	5.38	1	.017
+ Interaction with time	14	153700	153822	-76836	153672	4.23	1	<.001
+ Interaction with age category	17	154075	154223	-77020	154041	0.00	3	1
**Delayed recall models**								
Empty model	3	172261	172287	-86128	172255			
+ Covariates	9	168623	168701	-84302	168605	3650.60	6	<.001
+ Time effects	10	168228	168314	-84104	168208	397.10	1	<.001
+ Random slope for time	12	167548	167652	-83762	167524	683.86	2	<.001
+ Dog ownership as a predictor	13	167535	167648	-83755	167509	14.36	1	<.001
+ Interaction with time	14	167529	167651	-83751	167501	8.24	1	<.001
+ Interaction with age category	17	167873	168020	-83919	167839	0.00	3	1

Supplementary Table S4

Comparison of the cat ownership models.

	k	AIC	BIC	LL	Deviance	χ^2	*df*	p-value
**Verbal fluency models**								
Empty model	3	277968	277994	-138981	277962			
+ Covariates	9	275229	275307	-137606	275211	2750.40	6	<.001
+ Time effects	10	274679	274765	-137329	274659	552.81	1	<.001
+ Random slope for time	12	274296	274400	-137136	274272	386.79	2	<.001
+ Cat ownership as a predictor	13	274254	274367	-137114	274228	44.03	1	.001
+ Interaction with time	14	274231	274353	-137102	274203	24.47	1	<.001
+ Interaction with age category	17	274310	274458	-137138	274276	0.00	3	1
**Immediate recall models**								
Empty model	3	158737	158763	-79366	158731			
+ Covariates	9	154578	154656	-77280	154560	4171.59	6	<.001
+ Time effects	10	154003	154090	-76992	153983	576.30	1	<.001
+ Random slope for time	12	153676	153780	-76826	153652	331.77	2	<.001
+ Cat ownership as a predictor	13	153675	153787	-76824	153649	3.08	1	.079
**Delayed recall models**								
Empty model	3	172001	172027	-85997	171995			
+ Covariates	9	168284	168362	-84133	168266	3729.30	6	<.001
+ Time effects	10	167925	168012	-83953	167905	360.31	1	<.001
+ Random slope for time	12	167225	167329	-83600	167201	704.76	2	<.001
+ Cat ownership as a predictor	13	167223	167336	-83598	167197	3.88	1	0.049
+ Interaction with time	14	167208	167330	-83590	167180	16.59	1	<.001
+ Interaction with age category	17	167794	167941	-83880	167760	0.00	3	1

Supplemenetary Table S5

Comparison of the bird ownership models.

	k	AIC	BIC	LL	Deviance	χ^2	*df*	p-value
**Verbal fluency models**								
Empty model	3	243322	243348	-121658	243316			
+ Covariates	9	240959	241036	-120471	240941	2374.46	6	<.001
+ Time effects	10	240348	240434	-120164	240328	613.10	1	<.001
+ Random slope for time	12	240016	240119	-119996	239992	336.27	2	<.001
+ Bird ownership as a predictor	13	239993	240105	-119984	239967	24.60	1	<.001
+ Interaction with time	14	239995	240115	-119983	239967	0.51	1	.475
**Immediate recall models**								
Empty model	3	243322	243348	-121658	243316			
+ Covariates	9	240959	241036	-120471	240941	2374.50	6	<.001
+ Time effects	10	240348	240434	-120164	240328	613.10	1	<.001
+ Random slope for time	12	134620	134723	-67298	134596	105730.00	2	<.001
+ Bird ownership as a predictor	13	134591	134703	-67283	134565	30.93	1	<.001
+ Interaction with time	14	134593	134713	-67282	134565	0.54	1	0.462
**Delayed recall models**								
Empty model	3	243322	243348	-121658	243316			
+ Covariates	9	240959	241036	-120471	240941	2374.46	6	<.001
+ Time effects	10	240348	240434	-120164	240328	613.10	1	<.001
+ Random slope for time	12	134620	146672	-73273	146545	93783.25	2	<.001
+ Bird ownership as a predictor	13	134591	146649	-73256	146511	33.65	1	<.001
+ Interaction with time	14	134593	146658	-73255	146511	0.68	1	0.41

Supplementary Table S6
Comparison of the fish ownership models.

	k	AIC	BIC	LL	Deviance	χ^2	*df*	p-value
**Verbal fluency models**								
Empty model	3	240016	240042	-120005	240010			
+ Covariates	9	237709	237786	-118846	237691	2318.94	6	<.001
+ Time effects	10	237148	237233	-118564	237128	563.60	1	<.001
+ Random slope for time	12	236832	236943	-118404	236808	320.14	2	<.001
+ Fish ownership as a predictor	13	236831	236942	-118402	236805	2.75	1	.097
**Immediate recall models**								
Empty model	3	136968	136994	-68481	136962			
+ Covariates	9	133411	133488	-66696	133393	3569.26	6	<.001
+ Time effects	10	132857	132942	-66419	132837	555.89	1	<.001
+ Random slope for time	12	132570	132672	-66273	132546	291.22	2	<.001
+ Fish ownership as a predictor	13	132570	132681	-66272	132544	2.10	1	.015
**Delayed recall models**								
Empty model	3	148545	148570	-74269	148539			
+ Covariates	9	145375	145451	-72678	145357	3182.17	6	<.001
+ Time effects	10	145006	145092	-72493	144986	370.15	1	<.001
+ Random slope for time	12	144410	144513	-72193	144386	600.27	2	<.001
+ Fish ownership as a predictor	13	144412	144523	-72193	144386	0.01	1	.913
